# Supplementary material for: MetaRibo-Seq measures translation in microbiomes
Source: Nat Commun. 2020 Jun 29;11:3268. doi: 10.1038/s41467-020-17081-z (PMC7324362; doi:10.1038/s41467-020-17081-z)
Supplement: Supplementary file 10 — Supplementary Data 7 [file 41467_2020_17081_MOESM10_ESM.zip › File2/Confidence_High_Taxonomy/220690_out.krona.html]

Javascript must be enabled to view this page.

members
magnitude
magnitudeUnassigned
count
unassigned
taxon
rank

220690\_out

112

112
2
superkingdom

112
976
phylum

112
200643
class

order
171549
112

family
815
112

genus
816
112

457392

SRS147557\_contig\_number\_20684
1
species

species
1

SRS023829\_contig\_number\_10
821

species
818
1

SRS075773\_contig\_number\_3085

species
371601

SRS076804\_contig\_number\_11567
1

817

SRS011084\_contig\_number\_36495SRS012902\_contig\_number\_6SRS013098\_contig\_number\_49470SRS013158\_contig\_number\_4401SRS013215\_contig\_number\_232SRS013476\_contig\_number\_contig-100\_3127.171653SRS014459\_contig\_number\_contig-100\_235.227394SRS014613\_contig\_number\_19999SRS014736\_contig\_number\_1977SRS015190\_contig\_number\_965SRS015264\_contig\_number\_11641SRS015431\_contig\_number\_42093SRS015578\_contig\_number\_19452SRS015854\_contig\_number\_17442SRS015890\_contig\_number\_11885SRS015960\_contig\_number\_11220SRS016095\_contig\_number\_1192SRS016132\_contig\_number\_872SRS016267\_contig\_number\_2072SRS016495\_contig\_number\_11606SRS017433\_contig\_number\_17813SRS017521\_contig\_number\_29311SRS017622\_contig\_number\_395SRS018313\_contig\_number\_8982SRS018541\_contig\_number\_12227SRS018623\_contig\_number\_4332SRS018656\_contig\_number\_11677SRS018817\_contig\_number\_11829SRS018888\_contig\_number\_6118SRS018984\_contig\_number\_5864SRS019267\_contig\_number\_73SRS019601\_contig\_number\_15766SRS019787\_contig\_number\_862SRS020869\_contig\_number\_26574SRS021484\_contig\_number\_21861SRS022524\_contig\_number\_8301SRS023346\_contig\_number\_1745SRS023583\_contig\_number\_3997SRS023971\_contig\_number\_4634SRS024075\_contig\_number\_12207SRS024132\_contig\_number\_4028SRS024388\_contig\_number\_12482SRS024549\_contig\_number\_209SRS043411\_contig\_number\_contig-100\_343.103542SRS043701\_contig\_number\_877SRS046502\_contig\_number\_contig-100\_3734.70898SRS046712\_contig\_number\_180SRS048981\_contig\_number\_5532SRS049402\_contig\_number\_14010SRS050299\_contig\_number\_9592SRS051610\_contig\_number\_10738SRS053214\_contig\_number\_4532SRS053573\_contig\_number\_255SRS056259\_contig\_number\_21688SRS057717\_contig\_number\_2641SRS058723\_contig\_number\_4137SRS063040\_contig\_number\_33113SRS063127\_contig\_number\_1283SRS063190\_contig\_number\_10329SRS063489\_contig\_number\_6074SRS063985\_contig\_number\_22946SRS064276\_contig\_number\_30717SRS075021\_contig\_number\_3724SRS075398\_contig\_number\_725SRS075878\_contig\_number\_17613SRS077086\_contig\_number\_10131SRS077127\_contig\_number\_9521SRS077194\_contig\_number\_13836SRS077335\_contig\_number\_8200SRS077589\_contig\_number\_contig-100\_560.57799SRS077730\_contig\_number\_498SRS078419\_contig\_number\_35599SRS097920\_contig\_number\_18511SRS098571\_contig\_number\_33670SRS098644\_contig\_number\_34540SRS098655\_contig\_number\_1593SRS098881\_contig\_number\_15036SRS104036\_contig\_number\_7019SRS1041039\_contig\_number\_20948SRS1041145\_contig\_number\_23856SRS104311\_contig\_number\_11209SRS104485\_contig\_number\_2970SRS104636\_contig\_number\_20932SRS1054928\_contig\_number\_486SRS1055043\_contig\_number\_13108SRS1055069\_contig\_number\_6770SRS1055099\_contig\_number\_7080SRS140492\_contig\_number\_26143SRS140513\_contig\_number\_13259SRS142599\_contig\_number\_20189SRS143342\_contig\_number\_3191SRS143417\_contig\_number\_14288SRS143598\_contig\_number\_1292SRS144135\_contig\_number\_33331SRS144362\_contig\_number\_4069SRS144506\_contig\_number\_47758SRS146812\_contig\_number\_8154SRS147022\_contig\_number\_2750SRS147139\_contig\_number\_39695SRS147272\_contig\_number\_260SRS147377\_contig\_number\_11050SRS147653\_contig\_number\_6484SRS148091\_contig\_number\_302SRS148159\_contig\_number\_37454SRS149075\_contig\_number\_17121SRS893279\_contig\_number\_13380SRS893342\_contig\_number\_8613SRS971275\_contig\_number\_11067
108
species
